# Protein-Coding Region Derived Small RNA in Exosomes from Influenza A Virus–Infected Cells

**DOI:** 10.3390/ijms24010867

**Published:** 2023-01-03

**Authors:** Malgorzata Kwasnik, Wojciech Socha, Bartosz Czech, Magdalena Wasiak, Jerzy Rola, Wojciech Rozek

**Affiliations:** 1 Department of Virology, National Veterinary Research Institute, 24-100 Pulawy, Poland; 2 Biostatistics Group, Department of Genetics, Wroclaw University of Environmental and Life Sciences, 50-375 Wroclaw, Poland; 3 Department of Pathology, National Veterinary Research Institute, 24-100 Pulawy, Poland

**Keywords:** influenza A virus, exosomes, pcRNA, *RSAD2*, *CCDC141*, *IFIT2*

## Abstract

Exosomes may function as multifactorial mediators of cell-to-cell communication, playing crucial roles in both physiological and pathological processes. Exosomes released from virus-infected cells may contain RNA and proteins facilitating infection spread. The purpose of our study was to analyze how the small RNA content of exosomes is affected by infection with the influenza A virus (IAV). Exosomes were isolated by ultracentrifugation after hemadsorption of virions and their small RNA content was identified using high-throughput sequencing. As compared to mock-infected controls, 856 RNA transcripts were significantly differentially expressed in exosomes from IAV-infected cells, including fragments of 458 protein-coding (pcRNA), 336 small, 28 long intergenic non-coding RNA transcripts, and 33 pseudogene transcripts. Upregulated pcRNA species corresponded mainly to proteins associated with translation and antiviral response, and the most upregulated among them were *RSAD2*, *CCDC141* and *IFIT2*. Downregulated pcRNA species corresponded to proteins associated with the cell cycle and DNA packaging. Analysis of differentially expressed pseudogenes showed that in most cases, an increase in the transcription level of pseudogenes was correlated with an increase in their parental genes. Although the role of exosome RNA in IAV infection remains undefined, the biological processes identified based on the corresponding proteins may indicate the roles of some of its parts in IAV replication.

## 1. Introduction

Communication between cells within an organism is important both in physiological and pathological processes (e.g., the progression of cancer or the spread of viral infection). In recent years there has been a growing interest in the study of the mechanisms of pathogenesis of viral infections associated with intercellular communication. Extracellular vesicles (EVs) are released from the majority of cells into the extracellular space. Although they were initially treated as capsules designed to remove cellular waste, they are now understood to be message-carrying vesicles or multifactorial mediators of cell-to-cell communication. The influence of EVs on tissue repair [1,2], stem cell maintenance [3], communication in the central nervous system [4], cardiovascular disease [5,6], neurodegeneration [7], neoplastic processes [8] and inflammation [9] has been confirmed [10]. It has also been shown that EV composition changes during viral infection [11,12].

Exosomes are the smallest of the EVs and are approximately 30–150 nm in size. They perform an important function in transferring cargo molecules such as proteins, lipids, coding and non-coding RNAs, and DNA in intercellular communication to both close and distant target sites [13]. Exosomes contain proteins involved in RNA sorting, such as an endosomal sorting complex required for transportation (ESCRT), ALG-2 interacting protein-X (*ALIX*), and tumor susceptibility gene 101 (*TSG101*) [14,15]. They have been found in various body fluids, such as blood, urine, and saliva [13,16,17,18]. Exosomes have been found to stimulate immune responses by acting as antigen-presenting vesicles [13,19,20].

In exosomes, several RNA biotypes can be distinguished, including small non-coding RNA, messenger RNA (mRNA), ribosomal RNA (rRNA), and long non-coding RNA (lncRNA) [21]. The profile of RNA in the EV differs from the cellular RNA content, both in terms of biotypes and specific sequences, and can indicate the condition of the host cells [21]. Most previous studies on EV content have focused on small non coding RNA species: micro RNA (miRNA), Y-RNA, rRNA, transfer RNA (tRNA), piwi-interacting RNA (piRNA), small nuclear RNA (snRNA), and small nucleolar RNA (snoRNA) [22,23,24,25]. Larger types of RNA have also been described, such as mRNA, lncRNA, and circular RNA (circRNA) [23,24,26,27,28]. O’Brien et al. [21] reported finding “three types” of RNA in extracellular vesicles: confirmed functional RNA (e.g., intact mRNA and miRNA); intact RNA, potentially functional but not definitively confirmed to mediate intercellular communication (e.g., piRNA and vault RNA); and RNA fragments (fragments of tRNA, mRNA, or rRNA), some of which may be functional, and others non-functional products of degradation [21].

Exosomes have been found to carry antiviral elements and activate antiviral mechanisms [29]. They can also carry viral genomes, helping the virus spread by avoiding immune infiltration [30,31]. The association of EVs with a viral infection, such as human immunodeficiency virus (HIV), hepatitis B virus (HBV), hepatitis C virus (HCV), human papillomavirus (HPV), Epstein-Barr virus (EBV), human T-lymphotropic virus (HTLV), and herpesviruses have been studied [32,33,34]. It was observed that in response to viral infection, exosomes can enable the host to produce effective immunity by activating antiviral mechanisms and transporting antiviral factors between cells [35]. Viruses can also hijack the exosomal pathway to exploit cellular replication mechanisms and further spread infection. Studies on HIV-1, HTLV, Dengue virus (DENV), and HCV have shown that exosomes secreted from infected cells contain factors that regulate cellular response and spread infections to neighboring cells [31].

Infection with influenza viruses is a major concern to both human and animal health. Exosomes from cells infected with the influenza A virus (IAV) exhibited an increase in the level of some miRNA sequences that are known to inhibit influenza virus replication. Recently, Hong Y. et al. examined the expression of exosomal miRNAs in noninfected and HPAIV H5N1- Leghorn chickens. Most of the genes targeted by these differentially expressed miRNAs were found to be involved in the regulation of the MAPK signaling pathway, with several being identified as immune-related [36]. Additionally, it was proven that exosomes could carry viral components in a way that could be important for immune evasion but also as a mode of antigen transfer [34,37]. Although those studies proved the importance of exosomes in the course of IAV infection, they were limited to analysis of only a fraction of the exosome content and could give little attention to the transfer of RNA other than miRNA. The role of RNA contained in exosomes released from influenza virus–infected cells is still largely unknown. In this study, using high-throughput sequencing we have characterized the small RNA composition of exosomes derived from IAV-infected and mock-infected Madin–Darby canine kidney (MDCK) cells. Special attention was paid to differentially expressed protein-coding RNA (pcRNA) and pseudogenes.

## 2. Results

### 2.1. Peroxidase-Linked Assay

**Figure 1:**
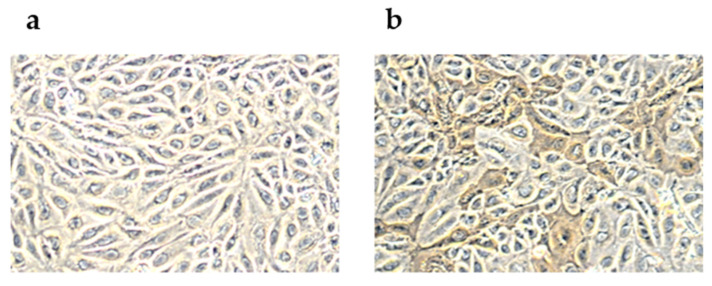
Immunoperoxidase staining of Madin–Darby canine kidney (MDCK) cells infected with equine influenza virus (MOI of 10), (**a**) Mock-infected MDCK cells; (**b**) MDCK infected with A/equi/Kentucky 81 cells.

A peroxidase-linked assay was used to show the multiplication of the equine influenza virus (EIV) in MDCK cells (Figure 1). The cells were inoculated with A/equi/Kentucky/81 (H3N8). After 1 h of adsorption cells were washed twice thoroughly with phosphate-buffered saline (PBS). After 24 h cells were fixed and stained using rabbit immune serum, horseradish peroxidase conjugate, and substrate. It was confirmed that the applied dose of the virus infects cells but does not disintegrate them and does not lead to their detachment from the surface.

### 2.2. Exosome Purification

**Figure 2:**
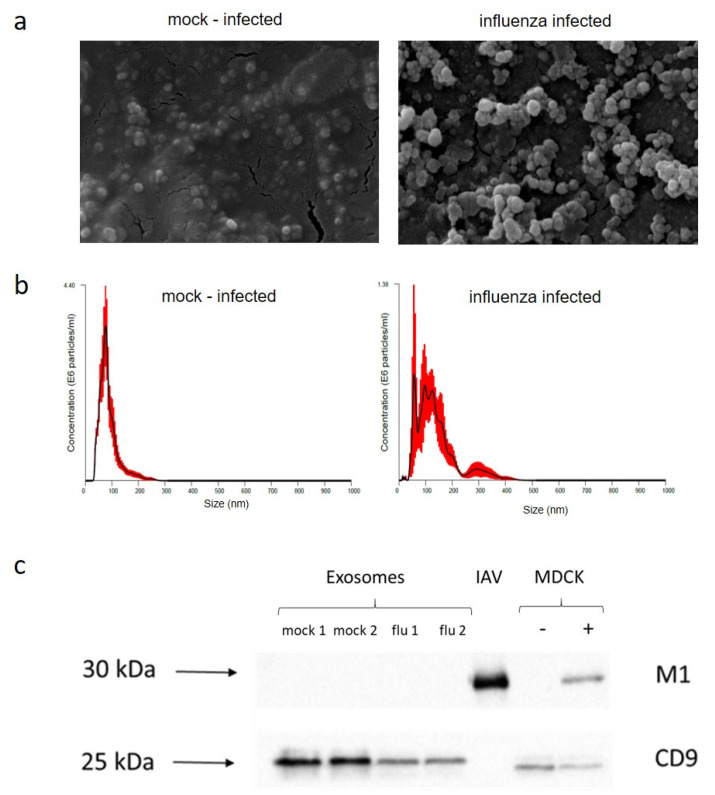
Identification and characterization of exosomes. (**a**) Scanning microscope photograph of purified exosomes. (**b**) Nanoparticle tracking analysis size distribution of exosomes isolated from mock-infected and influenza A virus (IAV)–infected MDCK cells. Red error bars indicate +/− 1 standard error of the mean. (**c**) Immunoblotting of exosomal marker (CD9) and IAV matrix protein (M1). Exosomes secreted by mock–infected (mock 1, 2) and IAV infected (flu 1, 2) cells; IAV–influenza virions; MDCK–mock–infected (−) and IAV infected cells (+).

Culture fluids after low-speed centrifugation were filtered (0.2 μm) and then influenza virions were removed by adsorption with chicken erythrocytes. The supernatants were ultracentrifuged. The isolation of exosomes from the medium of mock-infected and influenza-infected MDCK cells was monitored by scanning microscopy, nanoparticle tracking analysis (NTA), and immunoblotting analysis. Scanning microscopy revealed the presence of vesicles smaller than 200 nm, a size comparable to the 30–150 nm range assigned to exosomes [38] (Figure 2a). Similar results were acquired in NTA with the majority of nanoparticles exhibiting a size typical for exosomes. The mean size range for nanoparticles derived from mock-infected MDCK cells was 85 ± 6.4 nm, and this size was 140 ± 7.4 nm for the particles derived from influenza-infected cells. Nanoparticle concentration in the mock-infected cells was 1.89 ± 0.40 (E8 particles/mL) and in the IAV-infected cells was 0.84 ± 0.40 (E8 particles/mL) (Figure 2b). Immunoblotting confirmed the presence of CD9 marker protein in particles purified from both IAV-infected and mock-infected MDCK cell cultures (Figure 2c).

### 2.3. Sequencing Data Analysis

Totals of 51,779,137, 41,938,351, 46,630,234, and 81,467,933 sequence reads from exosomes isolated from mock 1 (mock-infected cell 1), mock 2 (mock-infected cell 2), flu 1 (influenza-infected cell 1) and flu 2 (influenza-infected cell 2) samples respectively were obtained after removal of low-quality reads and adapter sequences. For each of the analyzed samples over 80% of the reads were mapped to a reference dog genome (CanFam3.1, GenBank assembly GCA_000002285.2. For the flu 1 and flu 2 samples, 1.36% and 1.14% of the reads, respectively, were mapped to influenza virus A/equi/Kentucky/81 (H3N8) reference genome (CY028828.1–CY028835.1). Traces of reads mapped to the influenza virus genome were also observed in the control groups (mock 1, mock 2), which may have been due to the inaccuracy of the mapping software. (Table 1). We mapped the small RNA reads to the protein-coding regions of the influenza virus genome. Reads from all eight segments of the viral genome were found in exosomes secreted by infected cells (Table S2).

Proportions of different RNA biotypes were identified based on classification from the Ensembl v97 (July 2019) database [39]. In the case of transcripts annotated in Ensembl as miscellaneous RNA, the Rfam 14.8 database “https://rfam.xfam.org/ (accessed on 1 June 2022)” was used for identification based on RF number (RNA family). After filtering out sequences with low mean normalized counts, protein-coding RNAs represented the predominant category in each of the analyzed samples (Table 2).

**Figure 3:**
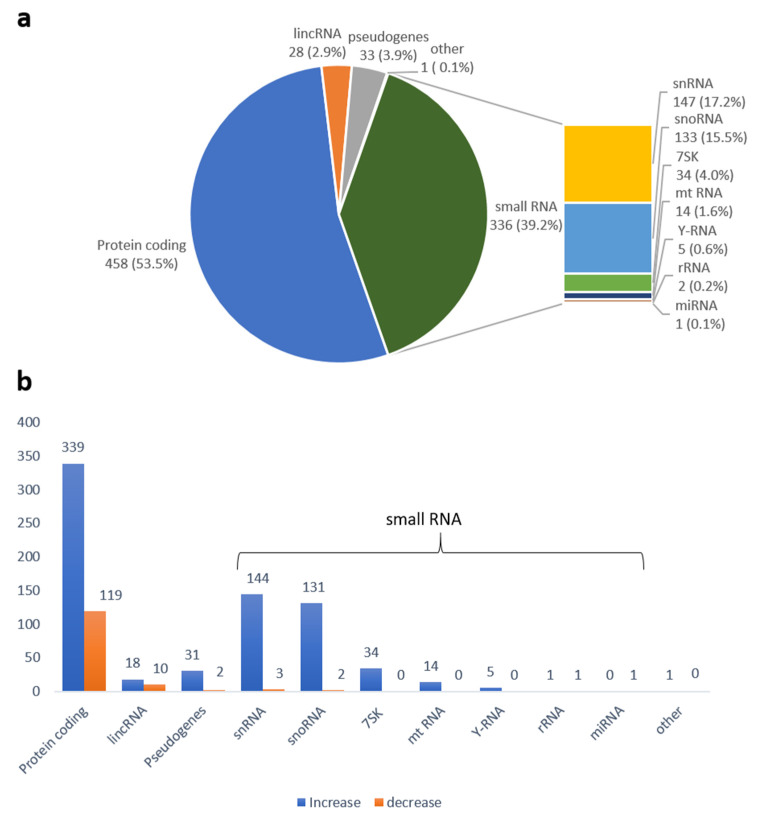
Differentially expressed RNAs identified in exosomes from influenza-infected cells, (**a**) Percentage of RNA biotypes; (**b**) Over- and underexpressed RNA biotypes, “other”—transcripts that could not be unambiguously assigned to any biotype.

As compared to mock-infected controls, 856 genes were significantly differentially expressed in exosomes from influenza-infected cells (flu 1, flu 2) (FDR < 0.05 and |log2FC > 2|). The analysis showed that 718 genes were upregulated while 138 were downregulated. Among the differentially expressed transcripts, the two largest categories were protein-coding, of which there were 458 transcripts, and small RNA (including snRNA, snoRNA, miRNA, Y-RNA, 7SK RNA, rRNA, and mtRNA), of which there were 336 (Table S1). A summary of the results is presented in diagram form in Figure 3.

**Figure 4:**
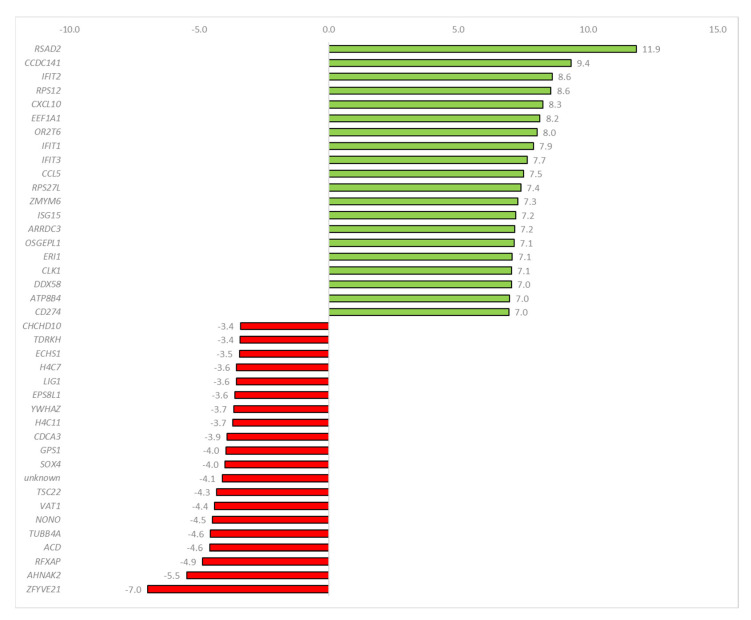
Protein−coding RNAs that exhibited the highest over− and underexpression in exosomes released from influenza−infected MDCK cells.

The protein-coding region-derived small RNAs with the highest expression variability were identified. The highest increase was observed for *RSAD2* protein–coding RNA (log2 fold change (FC) = 11.9), whereas the highest decrease was for Zinc Finger FYVE-Type Containing 21 (*ZFYVE21*) protein-coding RNA (log2FC = −7.0). A list of the 20 most overexpressed and 20 most underexpressed protein-coding RNAs is shown in Figure 4.

To identify the biological functions of differentially expressed pcRNAs, gene ontology analysis was performed using the functional analysis tool in Panther 17.0 “http://www.pantherdb.org/ (accessed on 1 June 2022)”. The biological function was assigned to 366 of the 428 differentially expressed individual pcRNA species. The three most commonly identified functional groups were associated with binding (gene ontology term (GO:0140657), catalytic activity (GO:0003824), and structural molecule activity (GO:0005198)) (Figure S1).

**Figure 5:**
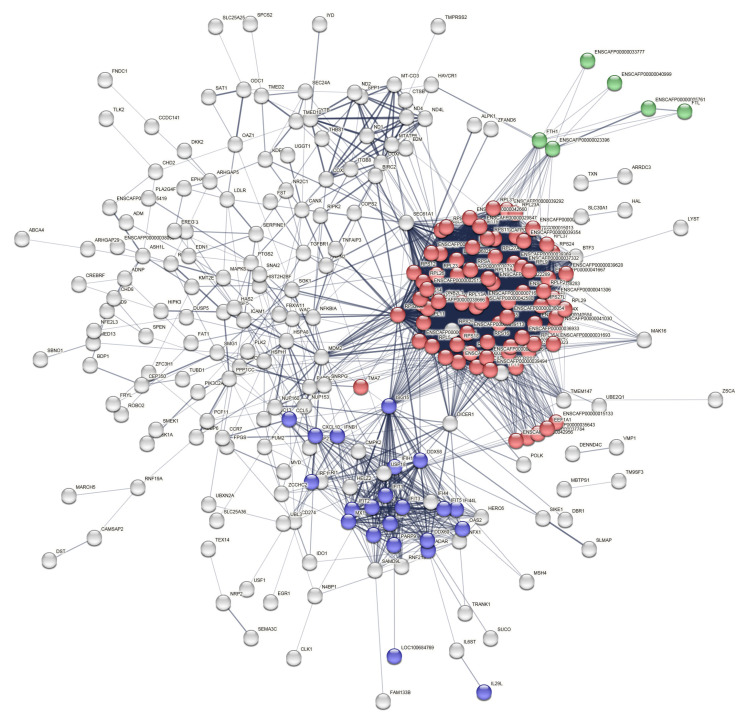
The interaction network of proteins encoded by transcripts overexpressed in exosomes isolated from IAV-infected MDCK cells, STRING pathway analysis. Colors indicate GO annotations: red—GO:0006412 “Biological process—Translation”; blue—GO:0009615 “Biological process—Response to virus”; green—GO:0006880 “Biological process—Intracellular sequestering of iron ion”; white—not assigned to a specific process.

Search tool for the retrieval of interacting genes/proteins (STRING) protein–protein interaction network analysis showed the clustering of 266 upregulated and 64 downregulated genes (pcRNAs). They were annotated into different functional categories. Among the 339 of which expression was increased, the most pronounced category was GO:0006412 “Biological process—Translation” (77 proteins, false discovery rate (FDR) = 5.65 × 10^−37^), GO:0009615 “Biological process—Response to virus” (22 proteins, FDR = 4.85 × 10^−9^), GO:0006880 “Biological process—Intracellular sequestering of iron ion” (6 proteins, FDR = 3.90 × 10^−4^) (Figure 5). Among the 119 pcRNAs with decreased expression, the largest categories were GO:0007049 “Biological process—Cell cycle” (20 proteins, FDR = 8.10 × 10^−3^) and GO:0006323 “Biological process—DNA packaging” (13 proteins, FDR = 1.05 × 10^−6^) (Figure S2).

As transcripts of pseudogenes could potentially interact with their parental genes, analyses of the correlations between the identified differentially expressed pseudogenes and their respective protein-coding transcripts were performed. In total, 33 differentially expressed pseudogenes transcripts as 31 over- and 2 underexpressed transcripts were identified in exosomes from influenza-infected MDCK cells. Both underexpressed pseudogenes were unprocessed, whereas six overexpressed examples were processed and 25 unprocessed. Pseudogenes with significant changes in expression were matched with their parental genes. A positive correlation was observed between the expression change of 23 identified pseudogenes and the respective protein-coding transcripts. Only in the case of LMF2 was overexpression of the pseudogene accompanied by a decrease in the expression level of the parental gene. For nine overexpressed pseudogenes no significant variability in the expression of their parental genes was observed (Table 3).

Protein–protein interaction network analysis of parental proteins of pseudogenes showed clustering of 19 out of 23 analyzed proteins. They were allocated to different functional categories with the most populated being GO:0010467 “Biological process—Gene expression” (19 proteins, FDR = 5.69 × 10^−14^) and GO:0006412 “Biological process—Translation” (16 proteins, FDR = 2.03 × 10^−16^) (Figure S3).

## 3. Discussion

For a reliable analysis of the content of exosomes, it is necessary to separate them from potential contaminants, including influenza virus particles, as they are in a similar size range. Therefore we applied a two-step protocol for exosome isolation combining ultracentrifugation with the removal of virions by adsorption on chicken erythrocytes [40]. The effectiveness of this method was monitored by scanning microscopy, NTA, and immunoblotting. A negative reaction for the viral *M1* protein contrasting with a positive one for the CD9 exosomal marker of purified exosomes indicated effective virion removal. After exosome purification, extraction of RNA, and its quality control, four libraries of influenza A–infected and mock-infected samples tested in duplicate were prepared. For each sample, over 80% of the reads were mapped to the *Canis lupus familiaris* reference genome. Additionally, around 1% of the reads in infected cells (flu 1, flu 2) were mapped to the IAV reference genome. This was in accordance with previous studies where both host and viral RNAs were detected in exosomes secreted from IAV-infected cells [41]. Our results showed that in addition to the presence of small RNAs (snRNA, snoRNA, miRNA, rRNA, mtRNA, 7SK RNA, and Y-RNA), exosomes are enriched with fragments of longer RNA sequences (pcRNA, pseudogene, and lincRNA). Previous research found the presence of multiple RNA biotypes in exosomes, including miRNA, piRNA, tRNA, snRNA, pcRNA, rRNA, and lincRNA [42,43,44]. We found fragments of pcRNA as the most abundant in exosomes both after influenza virus infection and in controls. Results obtained by Pérez-Boza et al. [45] suggested that besides an accumulation of small RNA species in exosomes, these vesicles may contain fragments of longer RNAs. Batagov et al. [46] found that exosomes contained 3′-end–derived mRNA fragments, while Mercer et al. [47] showed that RNA transcripts can undergo extensive post-transcriptional cleavage, generating a number of smaller coding and non-coding RNA species.

The analysis of our results showed 856 small RNAs differentially expressed in exosomes from influenza virus–infected cells. Most of them were upregulated (718), and the majority of these were derived from pcRNAs. Therefore, in our study, we focused on pcRNAs and related pseudogenes. However, because the preparation protocols for the libraries compiled in our experiments were designed for small RNA species, it was not possible to distinguish if the identified pcRNA, lincRNA, or pseudogenes represented fragmented or intact RNAs. Our results showed differences in pcRNA expression levels, we considered the potential significance of these transcripts in the context of the function of the proteins they encode. However, we are aware that these fragments can also function as regulators at the stages of both transcription and translation in the host as well as in target recipient cells.

Upregulation of pcRNA corresponding to proteins in the functional category GO:0006412 “Biological process—Translation” was observed in exosomes secreted from cells infected with IAV. This included 77 pcRNAs, mainly coding ribosomal proteins (RPs), e.g., *RPS12*, *RPS18*, *RPS6*, *RPS27*, *RPL4,* and *RPL18*, or translation elongation factors. Ribosomal proteins play an essential role in ribosome biogenesis, assembly, and translation as chaperones stabilizing rRNA species and promoting their correct folding in ribosomal subunits [48,49]. Moreover, RPs also have ribosome-independent functions: they regulate the cell cycle, apoptosis, cell proliferation, and tumorigenesis [49,50,51,52], and are involved in virus replication [52]. It was reported that after viral infections, translation of host mRNAs was often suppressed, but RP synthesis and ribosome biogenesis increased [53,54]. However, some studies have shown that in the transcriptional profile of peripheral whole blood samples from influenza-infected patients, the downregulated genes were significantly enriched for pathways related to gene translation ([55].

In fact, many RPs show an upregulation in virus-infected cells, e.g., *RPL4* in cells infected with EBV and *RPS27a* in cells infected with HBV [56,57]. It was reported that *RPS27*, the pcRNA of which was upregulated in exosomes in our study, played an important role in the replication of IAV, Drosophila C virus, DENV, HCV, Sindbis virus, and border disease virus (BDV) [58,59,60,61,62]. Ribosomal proteins L4 and *RPL18* have been identified as interacting with viral protein 3 of the infectious bursal disease virus (IBDV) [63,64]. Upregulation of pcRNA for RPs in exosomes may be a part of the mechanism of translation regulation during IAV infection. It is possible that RPs have alternative functions related to viral infection. Infected cells could also use exosomes carrying RP pcRNAs for intercellular communication, inducing anti-viral immunity or “switching” the translation machinery in recipient cells.

The second group of upregulated pcRNAs corresponded to proteins classified to category GO:0009615 “Biological process—Response to virus”, among which we identified pcRNA for interferon-stimulated genes (*ISGs*). Interferon (*IFN*) triggers a signal transduction cascade that leads to the activation of *ISGs* and induces the cellular antiviral defense mechanisms. Recently it has been confirmed that many viruses are able to upregulate the *ISGs* independently of the *IFN*, e.g., interferon regulatory factor 3 (*IRF3*) gene signaling. The pcRNA for viperin (*RSAD2*) has been identified in our study as the most upregulated in exosomes secreted by cells infected with IAV. Viperin participates in inhibiting RNA replication of viruses such as human cytomegalovirus, IAV, West Nile virus (WNV), DENV, HCV, HIV-1, Chikungunya virus (CHIKV), and ZIKA virus [65,66,67]. Viperin can inhibit the budding and release of the IAV virus by binding to farnesyl diphosphate synthase in lipid rafts [65,68]. It can also catalyze the conversion of cytidine triphosphate (CTP) to 3′-deoxy-3′,4′-didehydro-CTP, which can cause premature termination of RNA synthesis by the RNA-dependent RNA polymerase of some viruses [69]. Other significantly up-regulated pcRNA species belonging to the “Response to virus” biological function category included *IFIT1*, *IFIT2*, *IFIT3*, C-X-C motif chemokine ligand 10 (*CXCL10*), *ISG15*, *Mx1*, *Mx2*, DexD/H box helicase 58 (*DDX58*), or C-C motif chemokine ligand 5 (*CCL5*). All these genes were previously found to be upregulated after viral infection [55,70,71,72,73]. The interferon-induced proteins with tetratricopeptide repeat target viral protein synthesis by binding to eukaryotic translation initiation factor 3 subunit C (*eiF3c*) or subunit E (*eiF3e*) and suppressing translation initiation [74]. Imaizumi et al. [75] postulated that IFIT proteins may also increase the expression of *CXCL10*, which induces lymphocyte chemotaxis and may inhibit the replication of viruses. It was also shown that *DDX58* and *CXCL10* genes were induced after infection and significantly enriched in acute respiratory distress syndrome [76]. Our study showed that *DDX58* was also one of the most upregulated pcRNA species in exosomes during IAV infection. *Mx* proteins are dynamin-like GTPases exhibiting broad antiviral activity [77]. Verhelst et al. [78] postulated a model in which *Mx1* interacts with the influenza ribonucleoprotein complex and interferes with its assembly by disturbing the polymerase basic protein 2 (*PB2*)–nucleoprotein (*NP*) interaction. Interferon-stimulated gene 15 (*ISG15*) is a ubiquitin-like protein involved in the host antiviral response [79]. The antiviral effects of *ISG15* conjugation to targets (ISGylation) include blocking the entry and replication of different pathogens, DNA repair, autophagy, protein translation, and exosome secretion. It was shown that ISGylation inhibits exosome release, possibly preventing the spread of potential pathogens or protein aggregates without reducing the secretion of cytokines [80,81]. Villarroya-Beltri et al. [80] hypothesized that stress-activated ISGylation coordinates a cellular response by inhibiting translation and enhancing p53 activity. In our STRING protein pathway analysis, *ISG15* served as a link between translation- and antiviral response–related pathways. This indicates its multidirectional activity during IAV infection.

A small group of upregulated pcRNAs corresponding to proteins associated with the “Intracellular sequestering of iron ion” biological function was observed in exosomes from IAV-infected cells. Iron ions play key roles in DNA and RNA synthesis, mitochondrial respiration, and cell proliferation and differentiation [82]. Nevertheless, because of its capacity to promote the generation of reactive oxygen species, iron can be cytotoxic [83]. The disruption of the iron balance is associated with the severity of infections with viruses such as norovirus, DENV, HBV, WNV, HCV, bovine leukemia virus, and HIV [84,85,86,87,88,89,90]. One of the upregulated pcRNA corresponded to ferritin, which can exist in two forms: H-ferritin (*FTH*) and L-ferritin (*FTL*) [91]. In our study, the pcRNA species for both *FTH1* and *FTL* were upregulated in exosomes after IAV infection. Interestingly, Hailong Wang et al. [92] also previously confirmed lower iron and higher ferritin levels in patients suffering from H7N9 influenza infection.

The upregulation of pcRNAs associated with a viral infection and mostly related to ISGs was previously described. In the studies of Zabrodskaya, Y. et al., A549 cells were stimulated with exosomes derived from influenza-infected cells [41]. Estimation of the expression levels of selected ISGs in A549 cells showed a decrease of RIG1, MDA5, PKR, and IFIT1, but not of the antiviral gene MxA. The authors postulated that the simultaneous decrease in the expression of these genes indicates the immunosuppressive effect of exosomes. In our study, we demonstrated an upregulation of small RNAs derived from ISGs in exosomes from influenza-infected cells. Perhaps mRNA fragments transported by exosomes have a regulatory function and may affect gene expression in neighboring cells. The question remains whether the insertion of pcRNA fragments for antiviral proteins into exosomes promotes or inhibits viral infection.

The number of pcRNA species downregulated in exosomes was lower than the number upregulated and they mainly corresponded to proteins related to two processes: GO:0007049 “Cell cycle” (20 proteins) and GO:0006323 “DNA packaging” (13 proteins). Yuan He et al. [93] showed that replication of IAV induces cell-cycle arrest in the G0/G1 phase, which was observed as beneficial for the production of viral proteins. One of the proteins involved in the “Cell cycle” process is a cytoskeleton component, filamin A (*FLNA*), and the pcRNA for this protein was downregulated in this study. It was previously shown that IAV infection leads to dysregulation of *FNLA* expression, and resultantly to the activation of the JNK stress pathway, which helps to achieve efficient viral replication [94].

There was a group of downregulated pcRNA species corresponding to proteins classified into the “DNA packaging” category. The majority of these proteins are histones such as *HISTH1C*, *HISTH4C11,* and *HISTH4C16*. Histones are localized to the nucleus as a component of chromatin. In the extracellular form, they may act as antimicrobial, proinflammatory, and toxic agents [95]. Histone H4 was previously described as possessing a strong antiviral activity, which reduced IAV uptake by target cells [96]. Influenza virus genome replication and transcription take place in the nucleus of infected cells, with the significant role of the viral NS1 protein. By mimicking the histone sequences, NS1 gains access to histone-interacting transcriptional factors that may regulate inducible antiviral gene expression [97]. Whitfield et al. [98] reported that histone mRNA is rapidly degraded when DNA replication is inhibited. It may be reflected by the downregulation of histone pcRNA observed in exosomes in our study, and linked with previously reported cell-cycle arrest in the G0/G1 phase after IAV infection [93].

Differential analysis showed that the most downregulated in exosomes was pcRNA for *ZFYVE21*. This protein is a transcription factor with a zinc finger domain, which plays a significant role in gene regulation. Some regulating action of the *ZFYVE21* protein was found in respect of complement mediate inflammation in vivo [99]. Interestingly, this gene was previously observed to also be the most downregulated in the lungs of *Bordetella pertussis*-infected mice [100]. The protein-coding RNA for *AHNAK2* was the second most downregulated. Dong-Wei Wang et al. [101] confirmed that the knockdown of *AHNAK2* can lead to a decrease in cell proliferation, migration, and apoptosis, which is related to the inactivation of the mitogen-activated protein kinase–signaling pathway.

Since pseudogenes are closely related to parental genes, we highlighted a group of upregulated pseudogenes identified in the exosomes of influenza virus-infected cells. Pseudogenes are defined as gene variants that have lost their functions as a result of accumulated mutations. Although in the past they were regarded as transcriptionally silent “junk DNA”, in reality, a significant percentage of them undergo transcription, and their products may be involved in gene expression regulation [102,103]. Various potential regulatory mechanisms involving pseudogene transcripts have been proposed, such as processing material into small interfering RNA, acting as a decoy for transcription factors, acting as molecular sponges for microRNAs, or influencing chromatin or genomic architecture [103,104]. Depending on which mechanism is involved, the expression of pseudogenes could be positively or negatively associated with the expression of the pcRNA of their parental gene [105]. Differential analysis of pseudogenes by RNA-Seq is challenging and the results should be approached with caution. This is because the reliable allocation of genomic regions to pseudogenes remains difficult. In fact, short reads resulting from RNA-Seq techniques often contain too few sequence differences between the parental gene and its respective pseudogene to unambiguously assort them to one of these two categories [104]. Nevertheless, some characteristic patterns were observed in the differential expression analysis of pseudogenes in our study. The majority of the upregulated pseudogenes originated from genes associated with ribosomal functions and were associated with biological processes of translation and gene expression. In most cases, the increase in the transcription level of these pseudogenes was accompanied by a similar increase in their parental genes.

One of the genes for which this positive association was especially clear was ribosomal protein SA (*RPSA*), previously described as an important receptor for different respiratory tract pathogens and viruses and linked with multiple identified pseudogenes [106]. A similar positive association in a gene–pseudogene pair was observed in the case of a group of pseudogenes originating from the gene encoding eukaryotic translation elongation factor 1 alpha 1 (*EEF1A1*). The existence of multiple pseudogenes for *EEF1A1* in humans was previously observed, and their expression was found to be correlated with various diseases, mostly different types of cancers. Additionally, the involvement of *EEF1A1* pseudogenes was observed in CHIKV and hepatitis E virus (HEV) infection [107]. The role of *EEF1A1* pseudogene transcripts in influenza virus infection has not been studied before; however, this gene’s regulation in influenza-infected cells by circular RNA was previously described. It was found that expression of circ_0050463 circRNA in the cytoplasm of IAV-infected cells was increased, and through the mechanism of miRNA, sponges led to potentiated *EEF1A1* expression, which facilitated viral replication [108]. It is possible that a similar mechanism might be associated with the six *EEF1A1* pseudogenes identified in our study because for each of them higher expression was observed in influenza-infected cells.

Many of the pcRNAs derived from small RNAs upregulated in exosomes in our study corresponded to ribosomal proteins or were associated with the antiviral response of the cell. Correlation between EVs and cells for long RNA transcripts was described previously, suggesting that the EV content may be a snapshot reflecting the host cell condition [109]. However, the transport of mRNA in EVs was also described as a selective and targeted process [23,28,110]. Ribonucleic acids delivered by exosomes can play regulatory roles, acting as competing RNA affecting the stability, localization, and translational capability of mRNA in target cells [46]. The upregulated pcRNA detected in our study, the ribosomal proteins being a pertinent example, may influence the translation in the recipient cells. On the other hand, Xiaoyi Huang et al. proposed that the presence of fragments of long RNA could be explained by exosomes functioning as a reservoir for the removal of degraded mRNA and lncRNA [111]. In our research, we identified upregulated pcRNA-derived small RNAs for ribosomal proteins and proteins associated with response to viral infection. In contrast, downregulated pcRNA was associated with cell-cycle regulation and DNA packaging. It remains to be clarified whether these RNAs act by adapting the metabolism of a cell to viral infection or are part of the cell’s antiviral defense.

## 4. Materials and Methods

### 4.1. Peroxidase-Linked Assay

Peroxidase-linked assay (PLA) was performed as described previously [112]. A monolayer of MDCK cells on 96-well plates was inoculated with A/equi/Kentucky/81 H3N8 (1 h, 10 MOI). After 24 h of incubation at 37 °C, cells were washed, fixed, and incubated with serum from a rabbit immunized with A/equi/Kentucky/81 (HI titer 8024), horseradish peroxidase conjugate and SIGMAFAST™ 3,3′-diaminobenzidine substrate (Sigma-Aldrich, St. Louis, MO, USA). The results were analyzed under a microscope Nikon eclipse TS100 at a magnification of 40×, and pictures were taken with a Nikon D5000.

### 4.2. Cell Culture Inoculation

Madin–Darby canine kidney cells were cultured in Eagle’s medium containing 10% fetal bovine serum supplemented with Plasmocin (Invivogen, Toulouse, France). After a full-coverage monolayer was obtained, cells were rinsed twice with PBS. A/equi/Kentucky/81 (H3N8) was propagated in 10-day-old specific-pathogen-free (SPF) embryonated chicken eggs, titrated on MDCK cells, and used for inoculation. After 1 h of adsorption, the medium was removed, the cells were washed twice in PBS, and covered with Eagle’s medium without serum. Culture fluids were collected 24 h post-inoculation. In parallel, as a mock-infected control, culture fluids from uninfected MDCK cell cultures were collected. Both influenza-infected and mock-infected samples were prepared in duplicate.

### 4.3. Exosome Purification and RNA Extraction

Differential centrifugation was used for exosome isolation [113]. Culture fluids were centrifuged for 10 min at 300× *g* at 4 °C, then for 20 min at 2000× *g* at 4 °C. Supernatants were collected and filtered (0.2 μm). Subsequently, influenza virions were adsorbed using chicken red blood cells (0.2% final concentration). Red blood cells were removed by centrifugation for 10 min at 1370× *g* at 4°C. The hemagglutination test was used to monitor the adsorption process according to the WOAH manual [114]. Then supernatants were ultracentrifuged for 2 h at 164,243× *g* at 4 °C using an Optima L-110XP, with 70Ti rotor (Beckman Coulter, Krefeld, Germany) (Figure S4). The pellet was suspended in 0.5 mL of PBS and used for RNA isolation with miRNeasy Mini Kit (Qiagen, Hilden, Germany) according to the manufacturer’s instructions. The obtained material was taken through quantitative analysis with a Qubit™ RNA High Sensitivity (HS) Assay (Invitrogen, Carlsbad, CA, USA). The quality of the material was controlled through fluorescence-based quantification and RNA integrity number estimation using an Agilent 2100 Bioanalyzer (Agilent Technologies, Palo Alto, CA, USA). Influenza virions desorbed from chicken erythrocytes were concentrated by ultracentrifugation (3 h at 164,243× *g* at 4 °C using an Optima L-110XP, with 70Ti rotor) and used in western blotting.

### 4.4. Electrophoresis and Immunoblotting

Electrophoresis and immunoblotting were carried out as described previously [115]. We used the following samples: exosomes secreted by mock-infected MDCK cells, exosomes secreted by IAV-infected MDCK cells, preparation of influenza virions, mock-infected MDCK cells, and IAV-infected MDCK cells. Protein samples (20 µg) were subjected to SDS PAGE using a 12.5% polyacrylamide gel under non-reducing conditions (−DTT). Two monoclonal antibodies were used: against influenza A matrix protein (MCA401, Bio-Rad AbD Serotec, Puchheim, Germany) and human CD9 (MCA469GT, Bio-Rad Laboratories, Hercules, CA, USA). Then the appropriate conjugates were used, which respectively were Peroxidase AffiniPure Goat anti-mouse IgG H + L (AB_10015289, Cat. # 115-035-003; Jackson ImmunoResearch, West Grove, PA, USA) and Peroxidase AffiniPure Goat anti-rabbit IgG H + L (AB_2307391, Cat. # 111-035-144; Jackson ImmunoResearch, West Grove, PA, USA). The chemiluminescence signals were captured using a LAS3000 analyzer (FujiFilm Life Sciences, Stanford, CT, USA).

### 4.5. Nanoparticle Tracking Analysis

Quantification and size determination of exosomes was performed using a NanoSight NS500 instrument (Malvern Panalytical, Malvern, UK) by the Laboratory of Nanostructures of the Polish Academy of Science (Warsaw, Poland). NanoSight NTA 2.3 (build 0025) software (Malvern Panalytical) was used for the visualization and analysis of nanoparticles. Samples of exosomes derived from cell cultures were diluted 1:10 in PBS for optimal concentration before examination with the NTA system. The instrument operating temperature was set to 25 °C, and each sample was tested in 5 replicates.

### 4.6. Scanning Electron Microscopy

Pellets containing exosomes were fixed in a 2% EM-grade paraformaldehyde aqueous solution and then dehydrated in ethanol and acetone. Samples were mounted on a scanning electron microscopy (SEM) stage with carbon paste. To make the surface conductive, a coating of 2–5 nm gold–palladium alloy was applied by sputtering (Polaron SC7620; Quorum Technologies, Laughton, UK) using argon as the gas for plasma before imaging by SEM using a ZEISS EVO40 microscope (Carl Zeiss AG, Jena, Germany). Microscopy was performed under low beam energies (10.0 kV), mag = 50,000 KX.

### 4.7. High-Throughput Sequencing and Data Analysis

Libraries were prepared using SMARTer smRNA-Seq kit (Takara Bio, Shiga, Japan), which included: polyadenylation, cDNA synthesis, amplification, and purification. High throughput sequencing was performed on HiSeq2500 Illumina using a 2 × 100 read length by Macrogen Inc (Seoul, Republic of Korea). The resulting FASTQ date files with 100 bp reads were corrected in 3′ ends of the reads. Low quality reads were corrected using Trimmomatic v0.32, and adapter sequences were removed using CutAdapt v4.1 [116,117]. Next, reads were mapped to *Cannis lupus familiaris* (CanFam3.1) and influenza virus A/equi/Kentucky/81 H3N8 (CY028828.1–CY028835.1) reference genomes, using BWA-MEM v0.7.15. The resulting files were sorted, indexed, and compressed for further analysis. Mapped reads in BAM files were the subject of differential expression analysis using FeatureCounts software v2.10 and matched with transcript coordinates and functions based on reference genome annotations [118]. The results were corrected for False Discovery Rate (FDR) and normalized to RPKM (reads per kilobase of transcript per million reads mapped). Biotypes of all filtered reads were verified by mapping to Ensembl v97 (July 2019).

Differential expression analysis for IAV-infected and mock-infected cell cultures was performed using the DESeq2 R package with a linear model based on negative binomial distribution [119]. For each transcript logarithmic fold change (log2FC) was estimated. The significance of log2FC was tested using the Wald test. Multiple testing correction was applied using FDR. Only results with FDR < 0.05 and |log2FC| > 2 were considered significant. Gene ontology analysis of differentially expressed proteins coding mRNAs was performed for molecular function categories using PantherDB “http://www.pantherdb.org/ (accessed on 1 June 2022)”. The results with unknown annotations were not included in the analysis. The simplified workflow used for RNA analysis is presented in Figure S5.

## 5. Conclusions

In our study, we compiled a list of up- and downregulated RNA species contained in exosomes secreted by cells infected with IAV. To the authors’ knowledge, no such analysis had been presented prior to this. We observed differences in the regulation of pcRNA and pseudogenes fragments. While the role of RNA content in exosomes in IAV infection remains undefined, the indicated biological processes suggest directions for further research.

## Figures and Tables

**Table 1:** Numbers of reads mapped to *Canis lupus familiaris* (CanFam3.1, GCA_000002285.2) and influenza virus A/equi/Kentucky/81 H3N8 (CY028828.1–CY028835.1).

Sample	Total Filtered Reads	*Canis lupus familiaris*		Influenza A/equi/Kentucky 81	
		Reads	% of Total	Reads	% of Total
mock 1	51,779,137	43,271,948	83.57%	150	0.00%
mock 2	41,938,351	36,352,398	86.68%	2639	0.01%
flu 1	46,630,234	37,460,944	80.34%	632,383	1.36%
flu 2	81,467,933	69,411,940	85.20%	930,116	1.14%

**Table 2:** Total number of *Canis lupus familiaris* (CanFam3.1, GCA_000002285.2)–specific reads representing different biotypes.

Biotype	Mock 1	%	Mock 2	%	Flu 1	%	Flu 2	%
**Protein-coding**	6045	85.38	5809	85.38	6315	84.55	6350	84.63
**Pseudogene**	141	1.99	127	1.87	153	2.05	153	2.04
**lincRNA**	412	5.82	410	6.03	429	5.74	430	5.73
**small RNA**	482	6.81	458	6.73	571	7.64	569	7.58
snRNA	141	1.99	121	1.78	181	2.42	181	2.41
snoRNA	216	3.05	225	3.31	246	3.29	246	3.28
miRNA	14	0.20	15	0.22	15	0.20	15	0.20
rRNA	69	0.97	68	1.00	70	0.94	69	0.92
mtRNA	1	0.01	0	0.00	1	0.01	1	0.01
7SK RNA	28	0.40	17	0.25	44	0.59	43	0.57
Y-RNA	8	0.11	7	0.10	9	0.12	9	0.12
**Other ^a^**	5	0.07	5	0.07	6	0.08	6	0.08
**Total**	7080	100.00	6804	100.00	7469	100.00	7503	100.00

snRNA—small nuclear RNA; snoRNA—small nucleolar RNA; miRNA—microRNA; rRNA—ribosomal RNA; mtRNA—mitochondrial RNA; lincRNA—long intergenic non-coding RNA ^a^ transcripts that could not be unambiguously assigned to any biotype.

**Table 3:** Differently expressed pseudogenes matched with their respective parental genes.

Parental Gene Name	Pseudogene			Protein-Coding Transcript	
	Ensembl Id	log2FC	Type	Ensembl Id	log2FC
*LMF2*	ENSCAFG00000031971	7.036	U ^a^	ENSCAFG00000031984	−2.208
*RPL23A*	ENSCAFG00000024043	6.881	U	ENSCAFG00000015394	4.870
*RPS19*	ENSCAFG00000000177	6.813	U	ENSCAFG00000028485	3.205
*RPL12*	ENSCAFG00000003544	6.785	U	ENSCAFG00000020136	5.258
*EEF1A1*	ENSCAFG00000013990	6.762	U	ENSCAFG00000009708	8.151
*EEF1A1*	ENSCAFG00000017221	3.509	U		
*EEF1A1*	ENSCAFG00000002517	3.081	U		
*EEF1A1*	ENSCAFG00000002748	2.978	U		
*EEF1A1*	ENSCAFG00000031557	4.839	U		
*FTH1*	ENSCAFG00000001606	6.071	P ^b^	ENSCAFG00000015901	3.723
*RPS6*	ENSCAFG00000017909	5.896	U	ENSCAFG00000008776	4.971
*RPS6*	ENSCAFG00000014664	5.016	U		
*RPL21*	ENSCAFG00000029619	5.644	U	ENSCAFG00000015987	6.408
*RPL32*	ENSCAFG00000007159	5.549	U	ENSCAFG00000004198	5.967
*RPL32*	ENSCAFG00000008590	4.968	U		
*RPL32*	ENSCAFG00000018104	3.346	U		
*RPSA*	ENSCAFG00000016319	5.543	P	ENSCAFG00000005101	4.353
*RPSA*	ENSCAFG00000006064	4.191	U		
*RPSA*	ENSCAFG00000017317	5.249	P		
*RPL15*	ENSCAFG00000025328	5.342	U	ENSCAFG00000005764	2.109
*RPLP0P*	ENSCAFG00000009876	5.128	P	ENSCAFG00000010227	4.484
*RPL31*	ENSCAFG00000017238	4.322	U	ENSCAFG00000030034	4.237
*RPL19*	ENSCAFG00000004857	4.007	U	- ^c^	-
*RPL19*	ENSCAFG00000002237	3.371	U	-	-
*RPL4*	ENSCAFG00000018632	3.822	U	-	-
*RPS2*	ENSCAFG00000013905	3.580	U	-	-
*SNRPG*	ENSCAFG00000003361	3.346	P	ENSCAFG00000032595	6.411
*RPL10A*	ENSCAFG00000030323	3.211	U	-	-
*HSPA8*	ENSCAFG00000012507	2.885	U	ENSCAFG00000011666	2.231
*RPS26*	ENSCAFG00000011389	2.227	U	-	-
*UBA52*	ENSCAFG00000018652	2.107	P	-	-
*PSMA4*	ENSCAFG00000016728	−2.689	U	-	-
*CFl1*	ENSCAFG00000010275	−2.385	U	-	-

FC—fold change; ^a^—Unprocessed; ^b^—Processed; ^c^—No significant change in expression of those parental genes was observed.

## Data Availability

The raw data of the High Throughput Sequencing results have been submitted to NCBI Sequence Read Archive (SRA) under BioProject accession PRJNA904136.

## Supplementary Materials

The supporting information can be downloaded at: https://www.mdpi.com/article/10.3390/ijms24010867/s1.
