# Supplementary material for: Protein-Coding Region Derived Small RNA in Exosomes from Influenza A Virus–Infected Cells
Source: Int J Mol Sci. 2023 Jan 3;24(1):867. doi: 10.3390/ijms24010867 (PMC9820831; doi:10.3390/ijms24010867)
Supplement: Supplementary file 1 [file ijms-24-00867-s001.zip › Figures S1-S5, Table S1 S2 description.pdf]

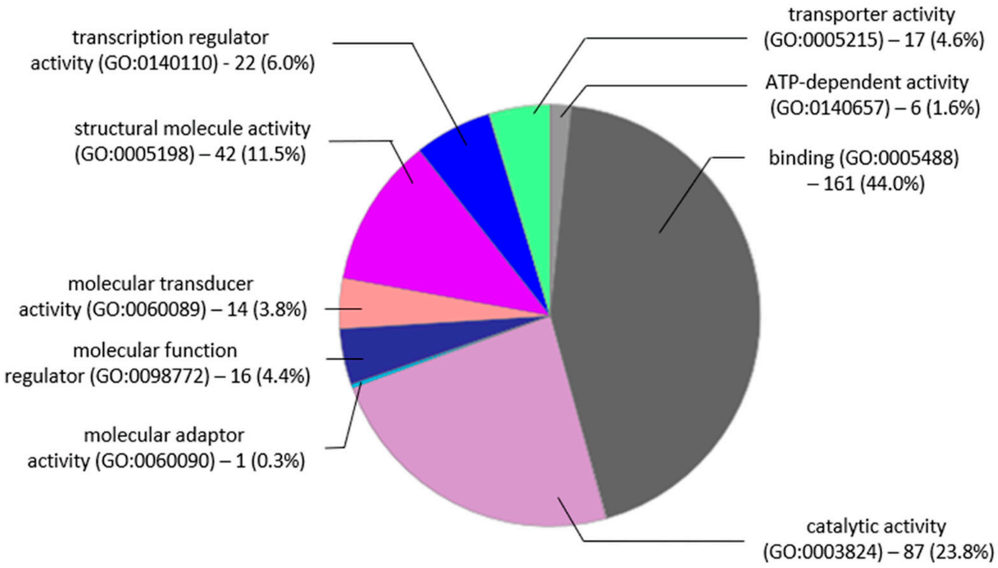


**Figure S1.** Functional analysis of differentially expressed protein-coding RNAs using Panther 17.0. ATP – adenosine triphosphate; GO – gene ontology term.

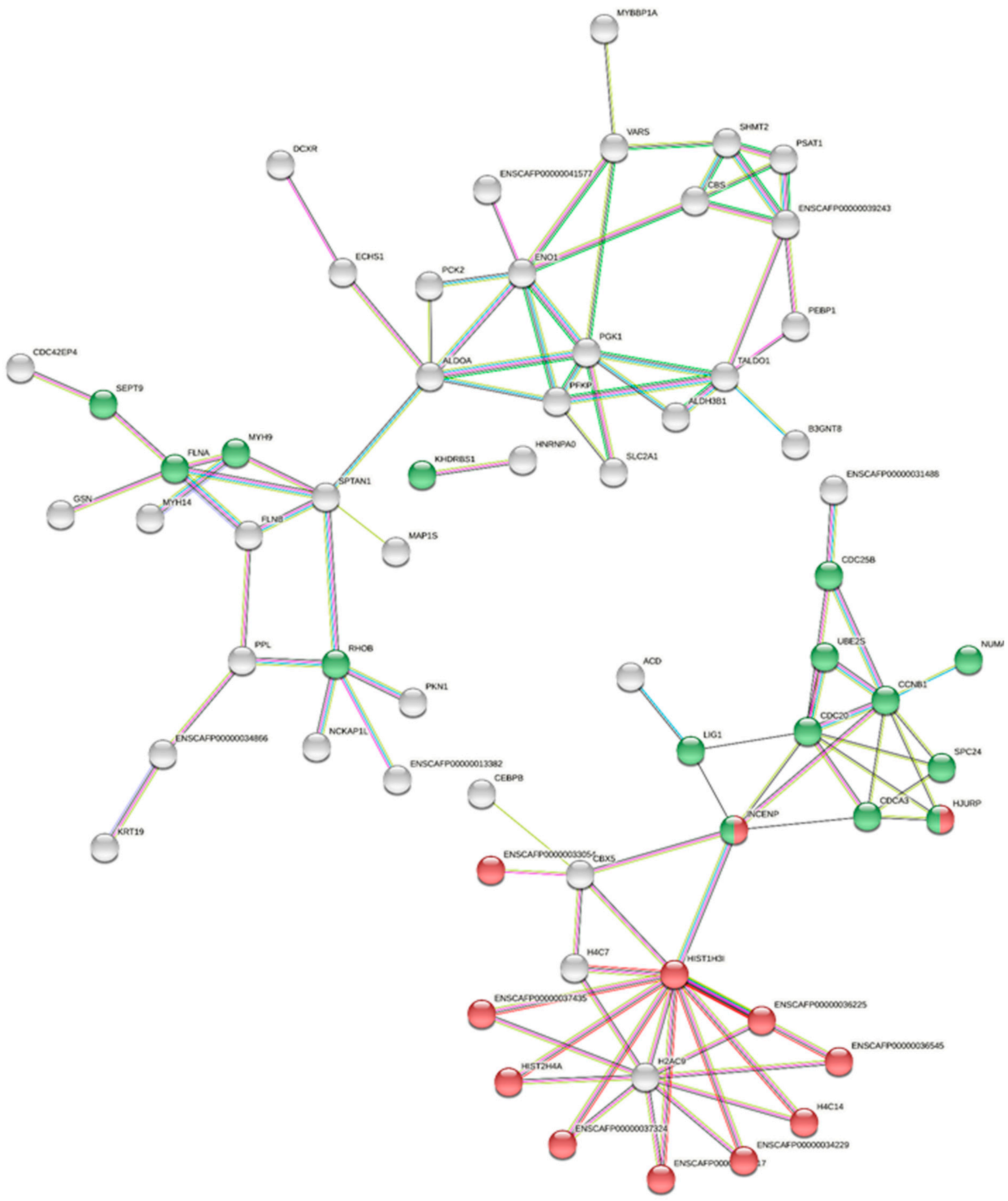


**Figure S2.** The interaction network of proteins encoded by transcripts underexpressed in exosomes isolated from IAV-infected MDCK cells, shown after STRING pathway analysis. Colors indicate GO annotations: green – GO:0007049 "Biological process – Cell cycle"; red – GO:0006323 "Biological process – DNA packaging".

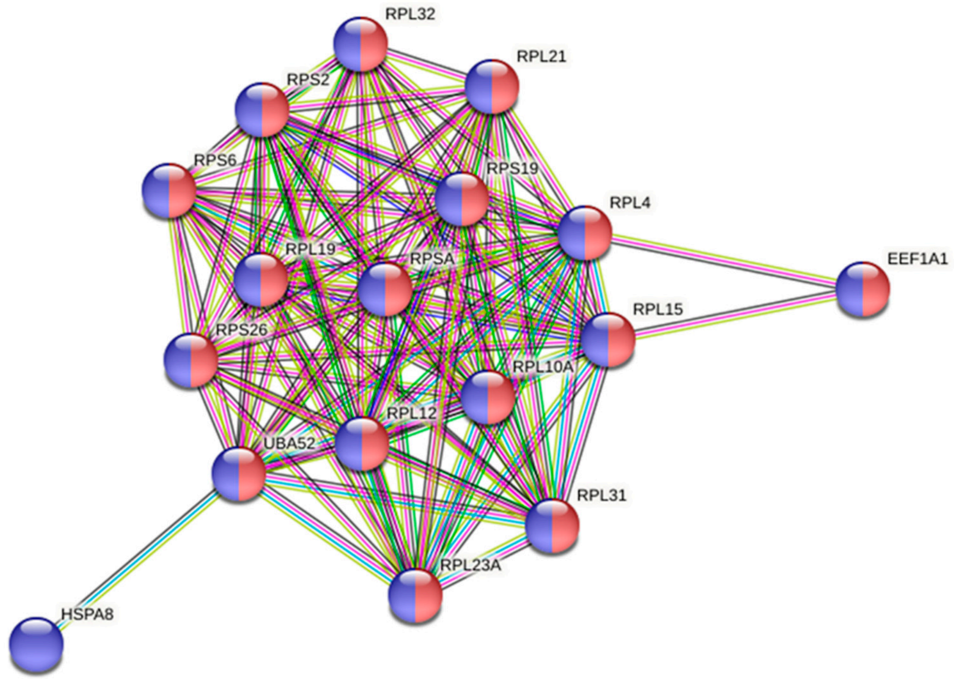


**Figure S3.** The interaction network of parental proteins of pseudogenes differentially expressed in exosomes isolated from IAV-infected MDCK cells, shown by STRING pathway analysis. Colors indicate GO annotations: red – GO:0006412 “Biological process – Translation”; blue – GO:0010467 “Biological process – Gene expression”.

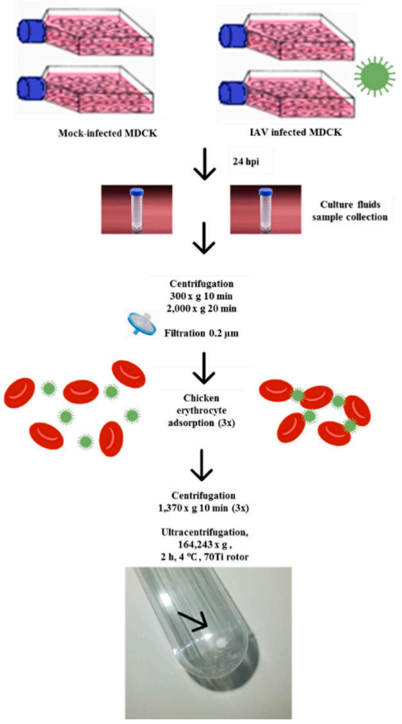


**Figure S4.** Overview of exosome isolation procedure, hpi – hours post inoculation.

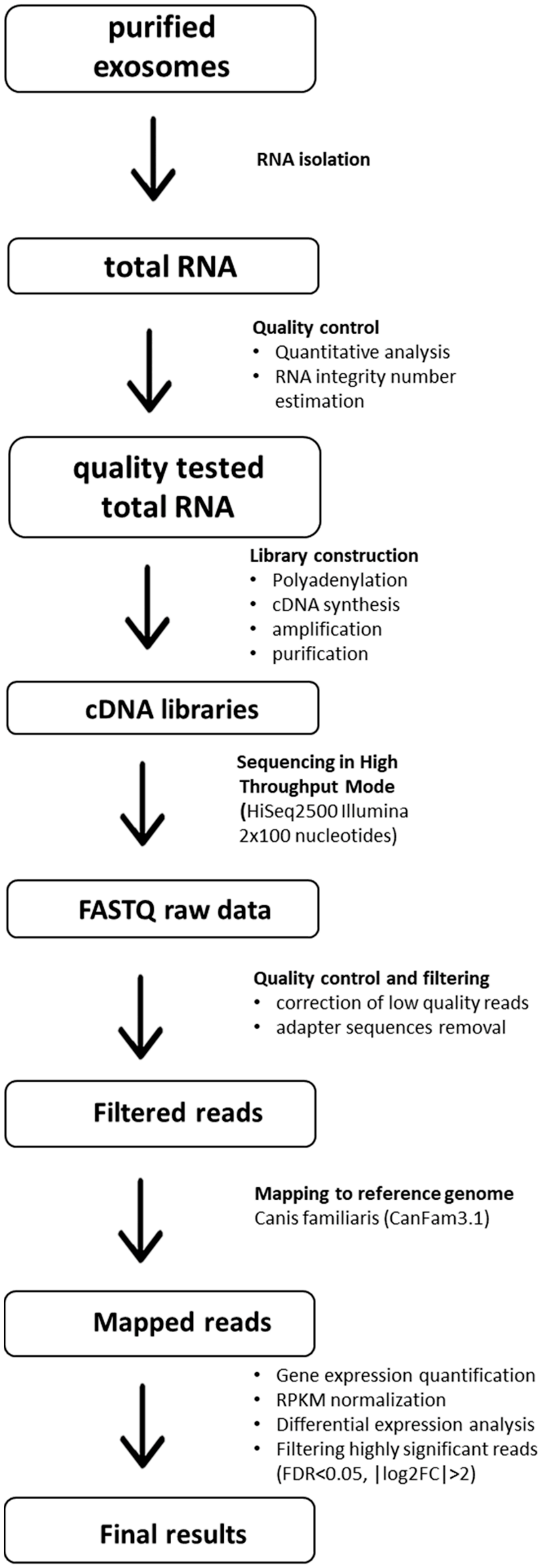


**Figure S5.** Overview of the analysis procedure of differentially expressed RNA in exosomes isolated from influenza-infected and mock-infected MDCK cells.
cDNA – complimentary DNA; RPKM – reads per kilobase of transcript per million reads mapped

**Table S1.** List of differently expressed RNAs in exosomes secreted by influenza infected cells

**Table S2.** Number of reads mapped to protein coding regions of influenza virus A/equi/Kentucky/81 reference genome (CY028828.1 - CY028835.1)
