# Supplementary material for: Protein-Coding Region Derived Small RNA in Exosomes from Influenza A Virus–Infected Cells
Source: Int J Mol Sci. 2023 Jan 3;24(1):867. doi: 10.3390/ijms24010867 (PMC9820831; doi:10.3390/ijms24010867)
Supplement: Supplementary file 1 [file ijms-24-00867-s001.zip › Table_S2.pdf]

Table S2. Number of reads mapped to protein coding regions of influenza virus A/equi/Kentucky/81 reference genome (CY028828.1 - CY028835.1)

Segment	Gene	Strand	Length	mock 1	mock 2	flu 1	flu 2
I	PB-2	+	2280	5	106	15906	35980
II	PB-1	+	2274	36	778	67751	108237
III	PA	+	2151	7	63	16066	25182
IV	HA	+	1701	5	62	45826	59073
V	NP	+	1497	10	99	33313	47703
VI	NA	+	1410	0	23	88915	115197
VII	M	+	982	0	31	7852	13589
VIII	NS	+	838	0	3	1498	3632
